# Temperature preference of Nile tilapia (*Oreochromis niloticus*) juveniles induces spontaneous sex reversal

**DOI:** 10.1371/journal.pone.0212504

**Published:** 2019-02-14

**Authors:** Renaud Nivelle, Vincent Gennotte, Emery Jules Kembolo Kalala, Nguyen Bich Ngoc, Marc Muller, Charles Mélard, Carole Rougeot

**Affiliations:** 1 Research and Education Center in Aquaculture (CEFRA), Liège University, Tihange, Belgium; 2 Laboratory for Organogenesis and Regeneration (LOR), Interdisciplinary research institute in the biomedical sciences (GIGA-I3), Liège University, Sart Tilman, Liège, Belgium; University of Illinois, UNITED STATES

## Abstract

Nile tilapia (*Oreochromis niloticus*) is an African freshwater fish that displays a genetic sex determination system (XX|XY) where high temperatures (above 32°C to 36.5°C) induce masculinization. In Nile tilapia, the thermosensitive period was reported from 10 to 30 days post fertilization. In their natural environment, juveniles may encounter high temperatures that are above the optimal temperature for growth (27–30°C). The relevance of the thermal sex reversal mechanism in a natural context remains unclear. The main objective of our study is to determine whether sexually undifferentiated juveniles spontaneously prefer higher, unfavorable temperatures and whether this choice skews the sex ratio toward males. Five full-sib progenies (from 100% XX crosses) were subjected to (1) a horizontal three-compartment thermal step gradient (thermal continuum 28°C– 32°C– 36.5°C) during the thermosensitive period, (2) a control continuum (28°C– 28°C– 28°C) and (3) a thermal control tank (36.5°C). During the first days of the treatment, up to an average of 20% of the population preferred the masculinizing compartment of the thermal continuum (36.5°C) compared to the control continuum. During the second part of the treatment, juveniles preferred the lower, nonmasculinizing 32°C temperature. This short exposure to higher temperatures was sufficient to significantly skew the sex ratio toward males, compared to congeners raised at 28°C (from 5.0 ± 6.7% to 15.6 ± 16.5% of males). The proportion of males was significantly different in the thermal continuum, thermal control tank and control continuum, and it was positively correlated among populations. Our study shows for the first time that Nile tilapia juveniles can choose a masculinizing temperature during a short period of time. This preference is sufficient to induce sex reversal to males within a population. For the first time, behavior is reported as a potential player in the sex determination mechanism of this species.

## Introduction

Sexual determination (SD) is a process by which a vertebrate decides which direction to take when it initiates its sexual differentiation as male or female [1]. SD allows for a sex ratio that is adapted to each species and crucial for population viability [2]. The nearly 36,000 known fish species exhibit a wide range of sex determining systems, ranging from strictly genetic (GSD), with sex chromosomes XX|XY or ZW|ZZ, to polygenic systems to environmental sex determination (ESD) [3,4]. The main ESD factor reported in fish is temperature (TSD). Since the first evidence of TSD in fish in Atlantic silverside, *Menidia menidia*, in 1981 [5], almost 60 species displaying TSD or genetic sex determination with a temperature effect (GSD+TE) have been described [6].

Nile tilapia (*Oreochromis niloticus*) is an African freshwater fish living in contrasting habitats. This species exhibits a complex sex determination system including 1) a genetic male heterogametic system (XX|XY) without morphologically differentiated sex chromosomes [7] 2) GSD polygenic factors [8] and 3) a masculinizing effect of high temperature (above 32°C) that can override the GSD [9–11]. Thermal treatments with temperatures above 32°C to 36.5°C for at least 10 days during the gonadal differentiation period (from 10 to 30 days post fertilization (dpf) for Nile tilapia) induce masculinization [11]. The magnitude of masculinization strongly depends on parental effects, from no effect to almost 100% males [7,11,12]. Exposure of juveniles to 36°C does not significantly affect survival rates [11] as the lethal thermal limit for Nile tilapia is 42°C [13]. Interestingly, an earlier period of thermal sensitivity was identified during embryogenesis (from 12 hours post fertilization to hatching) with a lower masculinization effect (from 6.0 to 26.7% males in 100% XX progenies) and survival rates at hatching (from 29.8% to 49.5%) [14]. It is now well known that high temperature has an effect on the cascade of sex differentiation, acting directly on the ovarian differentiation pathway and activating testis development [15]. Although the role of high temperature on sex determination is well described in Nile tilapia, the results are essentially reported under experimental conditions without any temperature choice. Only Bezault *et al*. (2007) reported naturally sex-reversed male specimens of Nile tilapia in Lake Volta, Ghana. At this location, temperatures in open water vary monthly from 27°C to 32°C throughout the year.

In the wild, Nile tilapia may encounter a wide range of temperatures with strong seasonal thermal variations, ranging from warm (28–34°C) to “cold” (22–26°C) seasons or extreme environments such as altitude lakes with constant “cold” temperatures (17–24°C) or hydrothermal hot springs (above 40°C) [12,16]. After spawning, females breed fry until 10 dpf, after which they are released in shallow water or flooded eulittoral grassland where high, potentially masculinizing temperatures (from 35 to 39°C) can occur [10,11]. Temperature is the most important abiotic factor controlling the metabolism of obligate poikilotherms, such as fishes [17,18]. One would expect the temperature preference to generally correspond to the optimal temperature for growth and/or other physiological functions [18,19]. In Nile tilapia, the optimal growing temperature in a controlled environment is approximately 27–30°C [20], below the masculinizing high temperatures above 32°C [21]. Thus, the relevance of thermal sex reversal in a natural context and the consequences for potential selective advantages are unclear. Additionally, it remains unclear whether undifferentiated *O*. *niloticus* juveniles would spontaneously move to high, potentially masculinizing temperatures, inducing sex reversal. The thermal preference of species exhibiting a GSD + TE sex determining system during the thermosensitive period for sex determination has only been studied in African catfish (*Clarias gariepinus*) [22]. These authors showed that undifferentiated juveniles migrated to high temperatures during the critical period of sex determination (6 to 8 days post hatching), inducing skewed sex ratios toward males (from 78 to 93%).

In this context, the aims of our study were to (1) identify the thermal preferendum of sexually undifferentiated *O*. *niloticus* juveniles during the thermosensitive period corresponding to gonadal differentiation and (2) evaluate the potential consequences on the sex ratio of the population.

## Materials and methods

### Ethics statement

This study was carried out in strict accordance with the European animal welfare recommendations and the guidelines of the Liège University Ethical Committee (CEFRA, LA 1610488). The protocol was approved by the Committee on the Ethics of Animal Experiments of the University of Liège (Protocol Number: 1747). All euthanasia was performed with an overdose (200 mg/L) of benzocaine (Sigma-Aldrich), and all efforts were made to minimize suffering. When fish exhibited eroded fins, lethargic behavior, swimming on the water surface or malformations, they were euthanized. Animal health and behavior were monitored at least 3 to 4 times during feeding. The duration of our experiment was 90 days per batch. A total of 4057 9-dpf juveniles were used. At the end of the experiment, 2008 fishes were sacrificed for sex identification. We reported that 243 fishes died naturally (almost 6% of the total initial number among a total of 19 batches), and no specific pathologies were observed.

### Progenies

**Fig 1:**
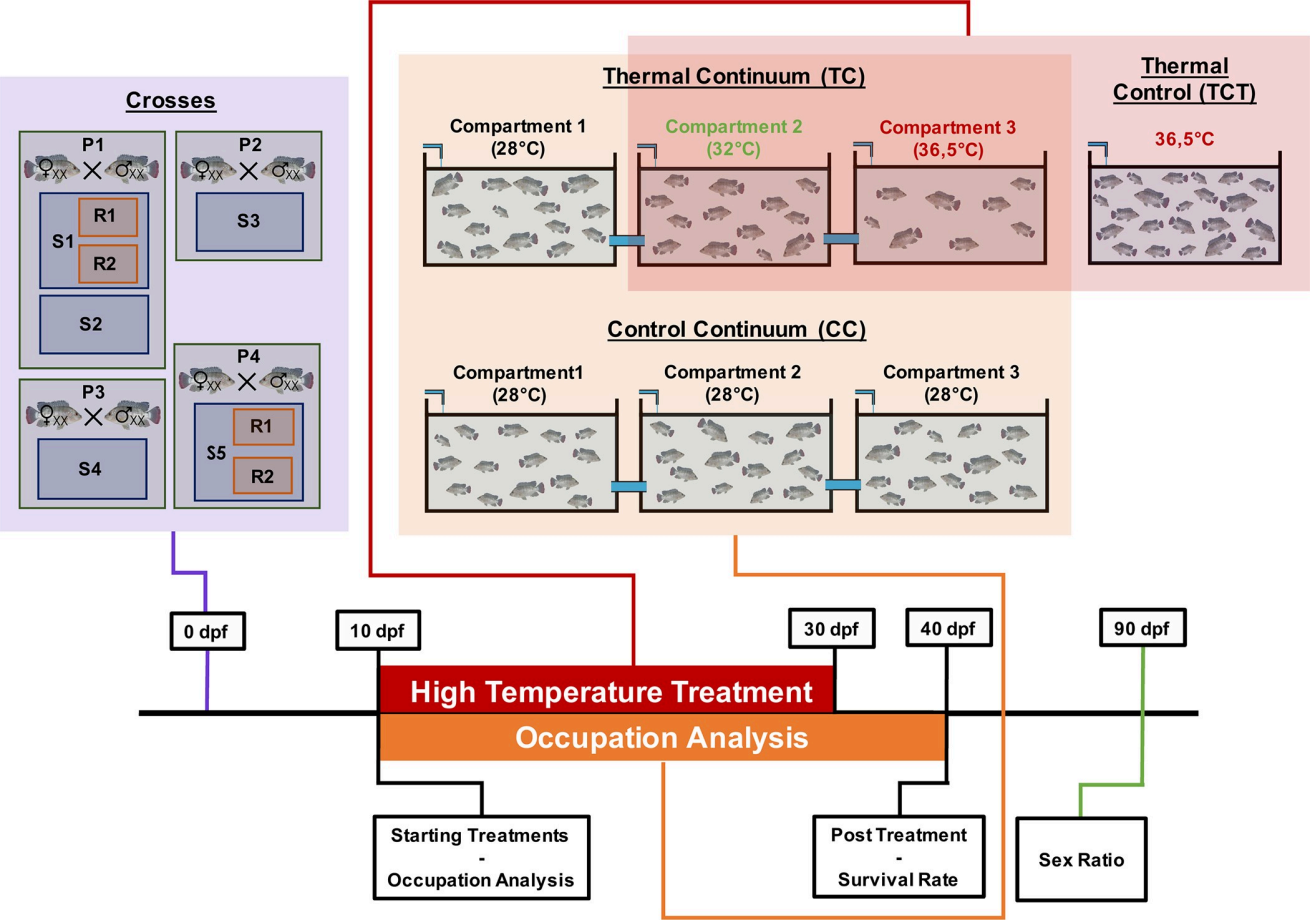
Experimental design from pairing to sexing. Five full-sib progenies (9 dpf) were individually used. The thermal continuum (TC) consisted of 3x50 L fish tanks at 28, 32 and 36.5°C connected by pipes; the control continuum (CC) consisted of 3x50 L fish tanks at 28°C connected by pipes; and the thermal control (TCT) consists of a unique 50 L tank at 36.5°C. The percentage of occupation was assessed from 10 to 40 dpf for each compartment. The temperature was increased within 3–4 hours (2°C/hour) at 10 dpf. High temperature treatment occurred from 10 to 30 dpf. The posttreatment survival rates and sex ratios were evaluated at 40 and 90 dpf, respectively.

Nile tilapias from the Lake Manzala strain were obtained from the Research and Education Center in Aquaculture (CEFRA), Liège University, Belgium. All matings were performed between XX females and XX males, the latter obtained by 17α-methyltestosterone (MT) hormonal sex reversal (50 mg 17α-MT/kg of feed from 10 to 40 dpf) [23]. XX progenies were used to better detect a potential masculinizing effect. Four couples of breeders (P1 to P4, Fig 1) were used to produce five full-sib progenies (S1 to S5). P1 produced 2 progenies (S1 and S2, respectively), and two spawns (S1 and S5) were sufficiently large to carry out an experiment in duplicate, named S1R1 and S1R2 and S5R1 and S5R2, respectively (Fig 1). Eggs were obtained by natural reproduction. Each female was isolated in a 250 L aquarium at 27°C. The spawning behavior of the female (genital papilla distension, nesting behavior) was observed using a local television system (CCTV) built with a Raspberry Pi connected to 4 different webcams. When courtship behavior occurred, the male was introduced into the aquarium. Once fertilization was complete, eggs were directly collected from the female mouth, and prophylactic disinfection was carried out (300 ppm hydrogen peroxide treatment for 30 minutes). After rinsing, eggs were incubated in Zug bottles at 28°C for 9 days.

### Experimental continuum

The experimental continuum was designed to observe the behavior of fish submitted to a horizontal thermal step gradient (continuum), as described by Santi *et al*. (2017). This facility was made up of 3 x 50 L fish tanks connected together by Plexiglas pipes (4.5 cm diameter, 15 cm in length, Fig 1). These pipes allowed the fish to freely move between compartments and were not connected in a linear way to prevent the direct migration of fish from one extreme compartment to another. Each compartment was equipped with thermostats and heating resistors (Biotherm 2000) to regulate the temperature of each compartment independently. Fish were exposed to a 12L:12D photoperiod during the whole experiment. Separate LED panels provided uniform lighting for each tank (1000 Lux).

For each progeny (S1 to S5), the experimental setup included a thermal continuum (TC), a control continuum (CC) and a thermal (36.5°C) control aquarium (TCT). Temperatures in the thermal continuum were 28°C (compartment 1), 32°C (compartment 2), and 36.5°C (compartment 3) from 10 to 30 dpf and 28°C from 30 to 40 dpf for all three compartments. A control continuum was set up just below the thermal continuum tanks to correct for asymmetric features in the room, where the temperatures were 28°C (compartment 1), 28°C (compartment 2), and 28°C (compartment 3) during the entire experiment. In addition, a separate 50 L thermal control aquarium was kept at 36.5°C from 10 to 30 dpf to measure the maximal sex reversal rate (thermosensitivity) of each progeny (S1 to S5, Fig 1).

### Experimental design

At 9 dpf, fry were harvested from the Zug bottles and counted. For each progeny, 150 fish were introduced into the TC and the CC. The initial number of fish in the TCT varied (n_i_ = 70 to 695), depending on the total number of juveniles counted at 9 dpf. In the continuums, fish were introduced into the left compartment (compartment 1), and the connecting pipes to the other compartments were unsealed at 10 dpf. Then, fish were free to move inside the continuum. Both thermal and control continuums were regulated at an initial temperature of 28°C. At 10 dpf, the temperature of the TC (compartments 2 and 3) and TCT was increased by 2°C/hour. The thermal treatment ended at 30 dpf when the temperature was decreased to 28°C at a rate of 2°C/hour. At 40 dpf, continuum batches were transferred into 50 L aquariums at 28°C for on growing until sex ratio analysis at 90 dpf.

### Rearing conditions

Fish were fed with a commercial tilapia diet close to satiation, and food rations were adjusted to ensure the same growth rate in all batches (47% proteins, 8% lipids, Coppens—The Netherlands). Food was manually distributed three times a day at the same time equally in each compartment of the continuum to avoid a correlation between occupation rate and food distribution. All tanks and continuum compartments were supplied with water from a recirculating system at 28°C. Fish were exposed to a 12L:12D photoperiod. The temperature was controlled with a precision mercury thermometer three times a day, and the oxygen concentration was maintained above 6 ppm. Water quality (pH, total ammonia nitrogen (TAN) and nitrite concentration) was assessed once a week.

### Continuum compartmental occupation analysis

Between 10 and 40 dpf (except for spawn S4, which we stopped tracking at 30 dpf due to transfer to other tanks), the occupation rate of TC and CC compartments was assessed by taking 2 photographs 3 times a day (morning (8 h– 10 h), midday (11 h– 13 h) and afternoon (15 h– 17 h)) before feeding. Fish in each photograph were counted using ImageJ software. Occupation rates were calculated for each compartment for each period (morning, midday and afternoon) of every day. Statistical analysis of compartmental occupation was performed by generating generalized linear models using the difference of the mean fish proportion in compartments TC and CC for all progenies as a dependent variable (DV), while time (dpf), compartment, period of the day, and interactions between these were used as independent variables [24]. Post hoc comparisons between compartmental occupations were obtained for each time unit, testing the null hypothesis that means are equals, using Tukey’s test within the "least-squares means" R package [25].

To evaluate differences in diffusion kinetics from the initial compartment 1 between TC and CC for all progenies, the Ti50 factor was set up. It corresponds to the time in days (dpf) at which half of the fish left the initial compartment 1 to diffuse across the continuum compartments. Ti50 was interpolated from 2^nd^ degree polynomial model curves fitted on the proportions of each progeny. Spearman’s correlation coefficient (ρ) between male percentages and the difference between Ti50 in CC and TC for each progeny were tested using Spearman’s test.

### Survival rate analysis

Survival rate posttreatment was evaluated at 40 dpf during the transfer of continuum batches into 50 L aquariums by counting the fish. Fish in TCT batches were also counted at 40 dpf. Analysis of survival rates was performed using the generalized linear model [26]. Post hoc comparisons for treatment survival rates of each progeny were assessed using Tukey’s test within the least-squares means R package [25]. At 40 dpf, the total biomass in TC and CC was also measured and divided by the total number of remaining fish to obtain the mean body weight in each continuum batch.

### Sex ratio analysis

At 90 dpf, a minimum of 50 fish per batch were randomly sampled and euthanized with an overdose (200 mg/L) of benzocaine (Sigma-Aldrich). The sex was determined using the aceto-carmine squash method for sexing juvenile fish [27]. A piece of gonad was removed, stained with a few drops of aceto-carmine, squashed with a cover slide and observed with an optical microscope (40× magnification). Phenotypic sex was assessed based on the presence of oocytes in the females and on the lobular morphology of the testis for the males. Analysis of the proportion of males was performed using the generalized linear model [26]. Post hoc comparisons between treatments for each progeny were assessed using Tukey’s test within the least-squares means R package [25].

### Statistical analysis

All statistical analyses were performed using R v3.4.4. The significant difference threshold was set at p-value ≤ 0.05. All plots were generated using the ggplot2 R package [28].

Spearman’s correlation coefficients (ρ) between survival rates, male percentages and means of fish proportion during the treatment period (from 10 to 30 dpf) inside each compartment of the thermal continuum were tested using Spearman’s test.

## Results

### Continuum compartmental occupation

**Fig 2:**
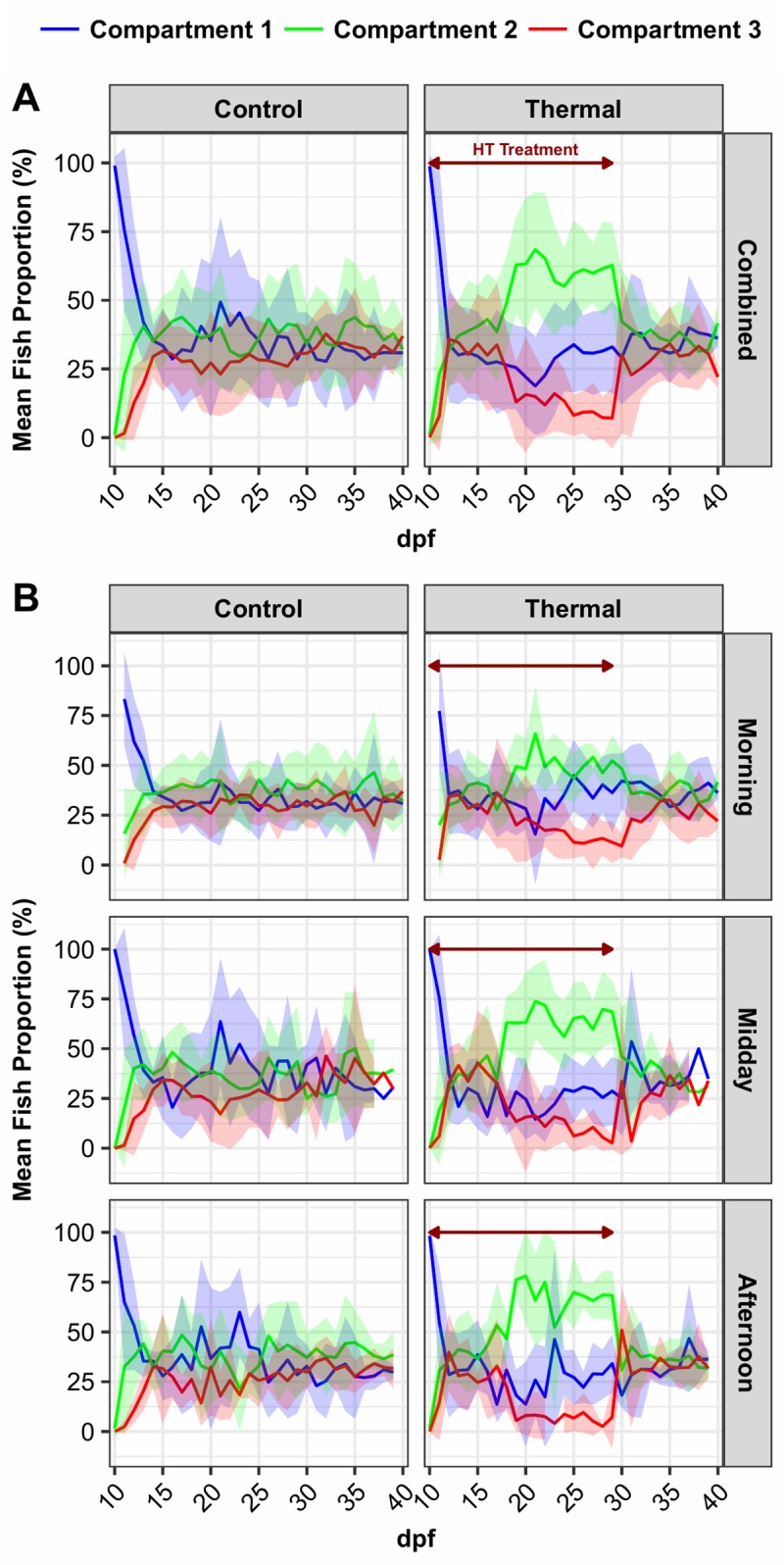
Mean fish proportion in thermal and control continuum compartments. Mean fish proportion (mean ± SD) for all progenies in control (CC, 28°C) and thermal continuums (TC compartment 1 (28°C), blue; compartment 2 (32°), green; compartment 3 (36.5°C), red) from 10 to 40 dpf. (A) Daily mean; (B) Distribution at 3 different periods of the day (morning, midday and afternoon). Red arrow: high temperature treatment period.

The general occupation pattern was different between CC and TC (Fig 2A). In CC, the mean fish proportion exhibited an initial diffusion during the first 5 days from compartment 1 to compartments 2 and then 3. After this period, fish tended to be equally distributed in CC with occupation values of 33.98 ±5.59%, 37.85 ±4.29% and 30.08 ±3.76% on average in compartments 1, 2 and 3, respectively, from 16 to 40 dpf. In TC, an initial diffusion step was followed by an unequal distribution of fish inside the continuum. During the high temperature (HT) treatment period, fish were mainly located in compartment 2 at 32°C (up to 60% on average from 19 to 29 dpf), while occupation of compartment 3 (36.5°) decreased from 34.13 ±18.21% (15 dpf) to 7.13 ±11.13% (29 dpf). Compartment 1 (28°C) exhibited an intermediate occupation pattern (27.91 ±4.46% on average from 15 to 29 dpf). After the HT treatment period, fish were equally distributed among the compartments, as in CC. However, fish distribution in CC and TC varied according to the period of the day (morning, midday and afternoon). Fish were equally distributed in the morning and moved to compartment 2 at 32°C at midday and afternoon (Fig 2B).

**Fig 3:**
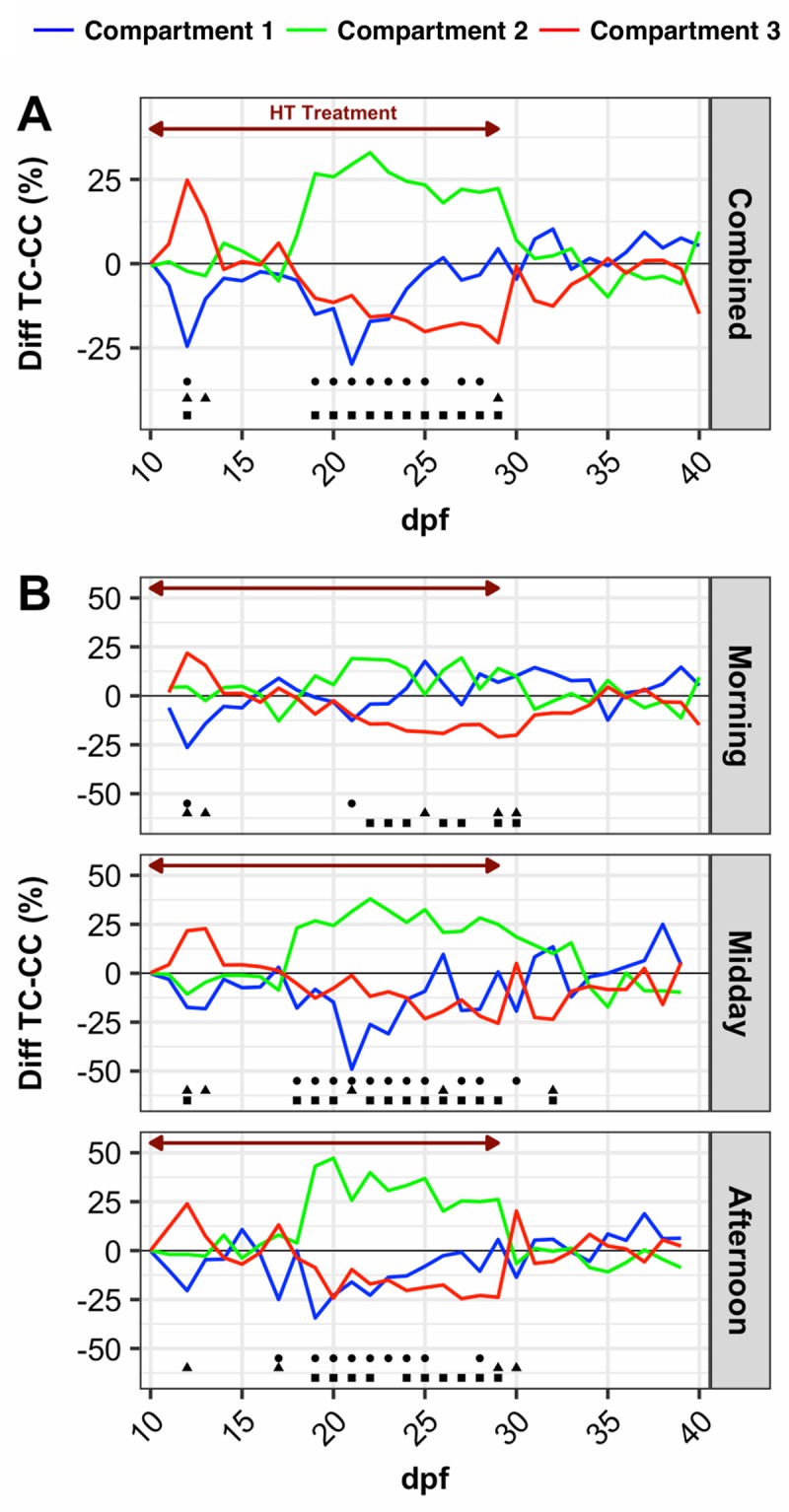
Normalized fish proportion in thermal continuum compartments. Difference of mean fish proportion in thermal continuum (TC, compartment 1 (28°C), blue; compartment 2 (32°), green; compartment 3 (36°C), red) from 10 to 40 dpf and the corresponding compartments in the control continuum (CC, 28°C) for all progenies. (A) Daily mean; (B) Distribution at 3 different periods of the day (morning, midday and afternoon). Red arrow: high temperature treatment period. Dots indicate statistically significant differences (p-value < 0.05) between ▪ compartment 2 –compartment 3, ● compartment 1 –compartment 2 and ▴ compartment 1 –compartment 3.

To better visualize the effect of the heat treatment, we normalized the proportion of fish in each compartment in TC by subtracting the proportion in the corresponding CC compartment (Fig 3A). Looking at these heat preference distributions, it appeared that the fish preferentially occupied the 36.5°C compartment 3 in TC compared to CC (up to 20% on average) during the initial days of the HT treatment. The difference in fish occupation between compartment 3 at 36.5°C and 1 at 28°C was statistically significant (p-value < 0.05). From 14 dpf and during the rest of the HT treatment period, occupation in compartment 3 at 36.5°C decreased compared to CC, and the most significant decrease occurred at 29 dpf. From 19 dpf to the end of HT treatment, compartment 2 (32°C) was clearly preferred (up to 25% on average). After treatment, no statistically significant difference (p-value > 0.05) between compartmental occupation was observed. The general pattern of normalized distribution varied depending on the period of the day, with midday and afternoon presenting a pattern more reminiscent of the global patterns (Fig 3B). The differences between TC and CC were stronger during midday and afternoon. In the morning, 14 significant differences (p-value < 0.05) were reported between compartmental occupation values, while 29 and 23 were found for midday and afternoon, respectively.

### Survival rates

**Fig 4:**
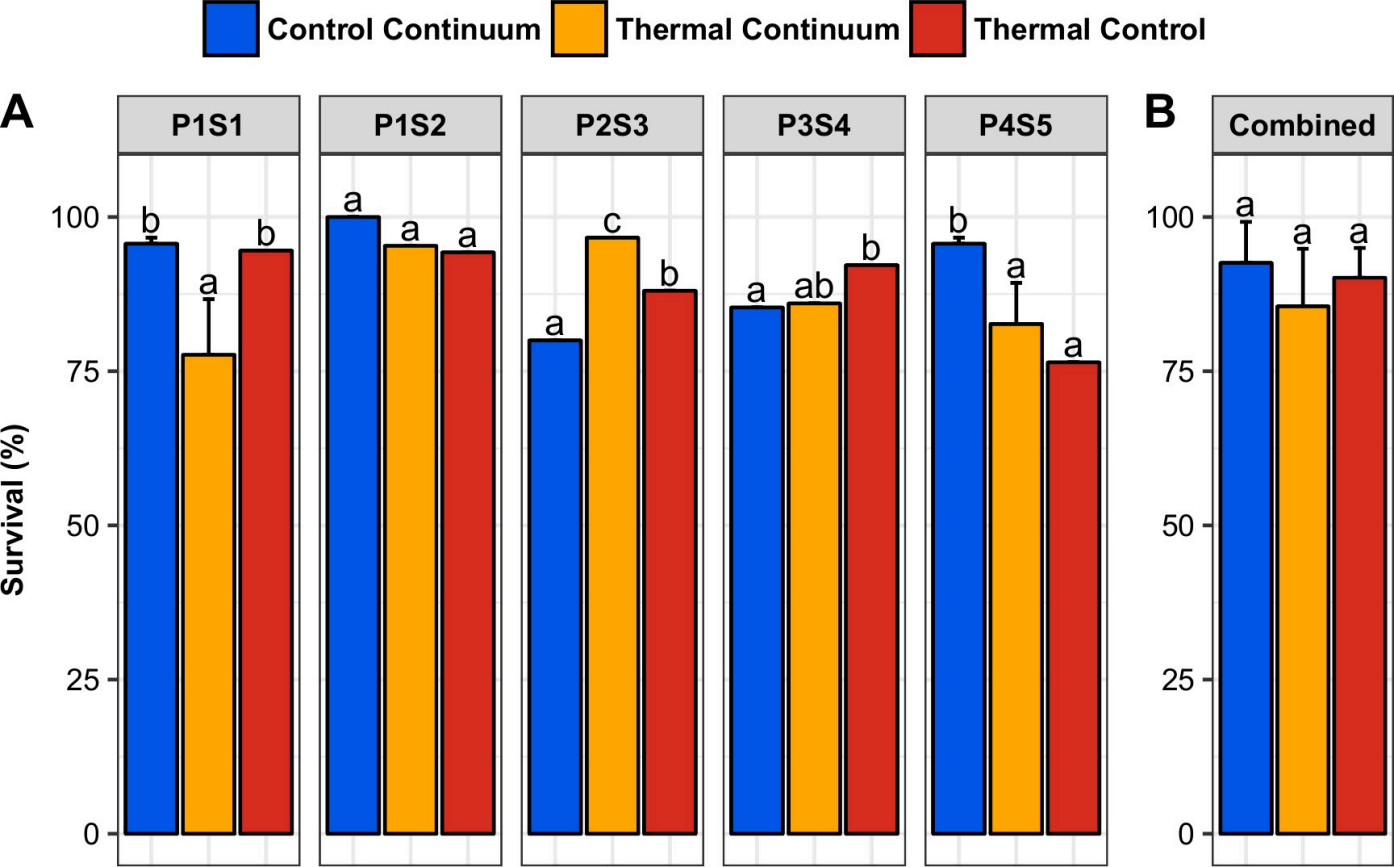
Posttreatment survival. Survival rates at 40 dpf (posttreatment) in the control continuum, thermal continuum and thermal control (A: by progeny; B: mean + SD). Within each chart, bars with the same letter are not significantly different (generalized linear model, p-value > 0.05).

The posttreatment mean survival rates (40 dpf) were 92.6 ±6.6%, 85.5 ±9.3% and 90.2 ±4.8% in CC, TC and TCT, respectively (Fig 4B). These survival rates remained within an acceptable range, and no significant difference occurred between treatments (Fig 4A, S1 Table). No difference in growth occurred between TC and CC at 40 dpf (1.33 ±0.40 g in CC and 1.35 ±0.16 g in TC).

### Sex ratios

**Fig 5:**
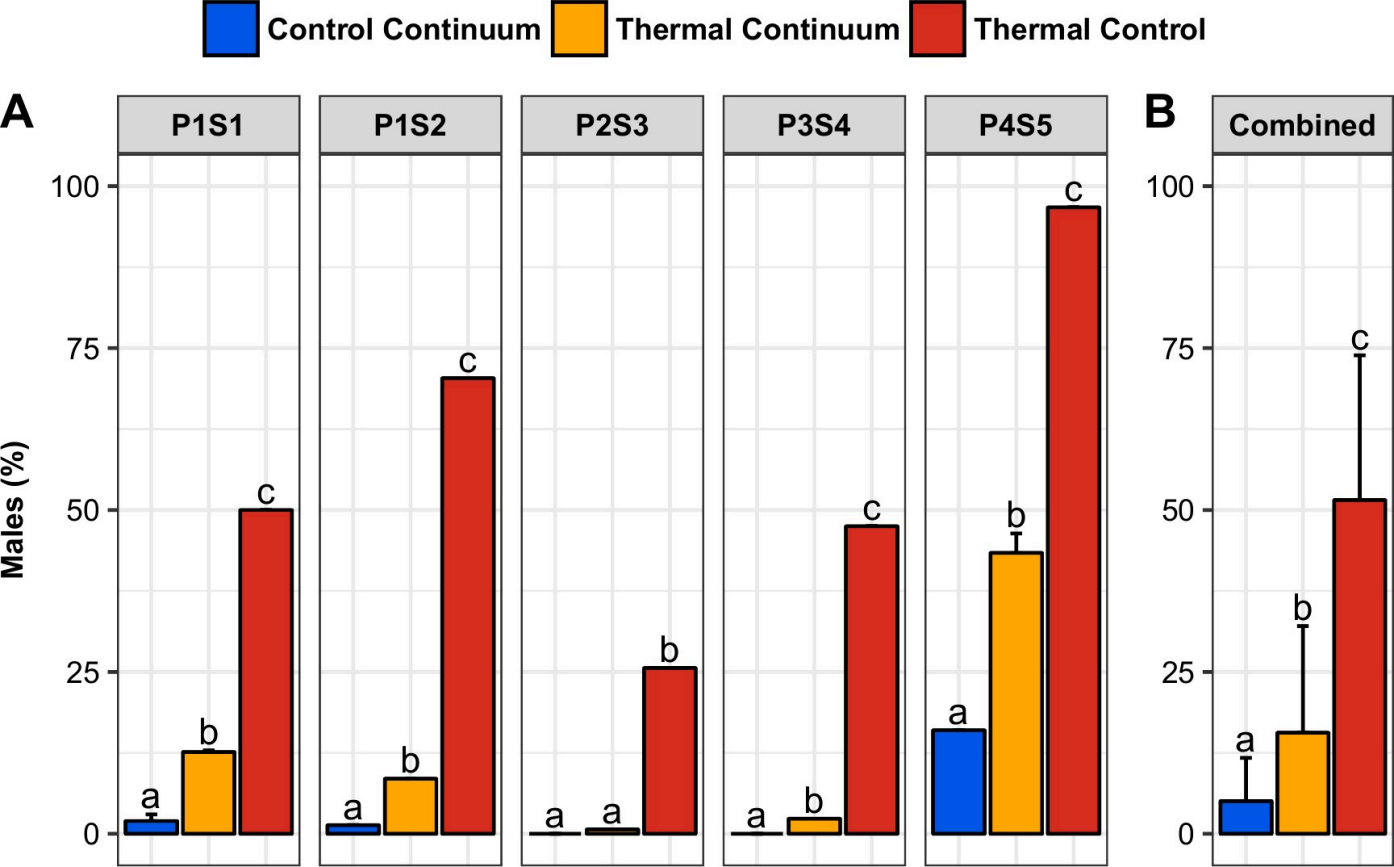
Sex ratios. Sex ratios (percentage of males) at 90 dpf in the control continuum, thermal continuum and thermal control (A: by progeny; B: mean ± SD). Within each chart, bars with the same letter are not significantly different (generalized linear model, p-value > 0.05).

The mean percentages of males (90 dpf) in CC, TC and TCT were 5.0 ±6.7%, 15.6 ±16.5% and 51.5 ±22.3%, respectively and were significantly different from each other (p-value < 0.05) (Fig 5B, S1 Table). The proportion of males in the CC ranged from 0.0% to 16.0% (Fig 5A). The thermosensitivity of all tested progenies was demonstrated by the percentage of males recorded in the 36.5°C control (TCT). Sex ratios ranged from 25.6 to 96.7% and were statistically (p-value < 0.05) higher than values observed in the 28°C control (CC). In all progenies, the sex ratio was significantly (p-value < 0.05) skewed toward males in the TC, with percentages of males ranging from 2.3 to 43.4%, except in S3 (0.7% males). No differences in sex ratios were observed between the replicate progenies P1S1R1/R2 and P4S5R1/R2 or between the replicate progenies P1S1 and P1S2 (p-value > 0.05) (Fig 5A).

### Correlations between proportion of males and survival rate

Male percentage values in CC, TC and TCT were positively correlated (0.85 < ρ < 0.98, p-value < 0.01) (Table 1). Comparison between percentage of males and survival rates showed a significant correlation in CC and TC (ρ = 0.67, p-value < 0.05 and ρ = -0.91, p-value < 0.001, respectively). No significant correlation between percentage of males and survival rate was observed in TCT.

**Table 1:** Correlation matrix between male proportions and survival rates.

	% Males CC	% Males TC	% Males TCT	% Survival CC	% Survival TC	% Survival TCT
**% Males CC**				**0.67** *		
**% Males TC**	**0.98** ***				**-0.91** ***	
**% Males TCT**	**0.85** **	**0.87** **				**0.29**

Spearman correlations coefficient matrix (Rho values) of survival rates (%) and male proportions (%) according to treatments (CC: control continuum, TC: thermal continuum and TCT: thermal control). Statistical significance

*** < 0.001

** < 0.01

* < 0.05.

### Correlations between progeny behavior in TC, sex ratio and survival rate

Male percentage in TC was negatively correlated with the mean fish occupation of compartment 1 (28°C) during the treatment period (ρ = -0.73, p-value < 0.05) (Table 2). A positive correlation between male proportion and compartment 2 (32°C) occupation was detected (ρ = 0.93, p-value < 0.001), but no significant correlation between compartment 3 (36.5°C) occupation and male percentage was observed. Survival rates in TC were significantly negatively correlated only with compartment 2 occupation (ρ = -0.98, p-value < 0.01).

**Table 2:** Correlation matrix between fish occupation in TC and male proportion and survival.

	Compartment 1 occupation	Compartment 2 occupation	Compartment 3 occupation
**% Males TC**	**-0.73** *	**0.93** ***	**-0.24**
**% Survival TC**	**0.60**	**-0.98** ***	**0.47**

Spearman correlation coefficient matrix values (Rho values) of survival rates (%) and male proportions (%) in thermal continuum (TC) according to the average fish proportion during the treatment period (from 10 to 30 dpf) in compartments 1 (28°C), 2 (32°C) and 3 (36.5°C). Statistical significance

*** < 0.001

** < 0.01

* < 0.05.

### Correlation between sex ratio and diffusion coefficient Ti50

**Fig 6:**
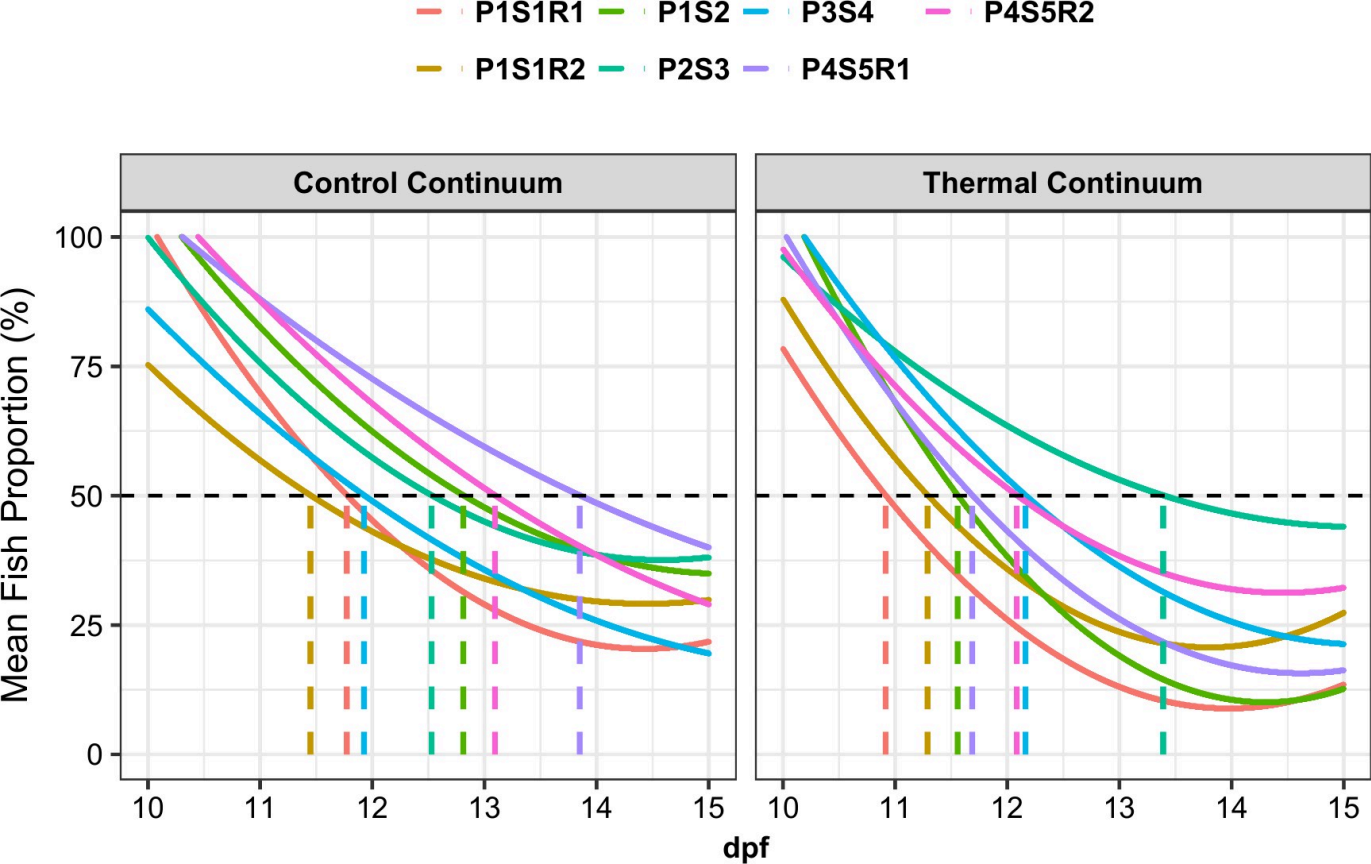
Initial diffusion step in thermal and control continuums. Initial fish diffusion from compartment 1 to compartments 2 and 3 in control (28°C– 28°C– 28°C) and thermal (28°C– 32°C– 36.5°C) continuums for each progeny. Curves were modeled using a 2^nd^ degree polynomial model to obtain days (dpf) at which 50% of fishes left the initial compartment 1 (Ti50 value, dashed color lines).

Ti50 values representing fish diffusion kinetics from compartment 1 in TC and CC varied between progenies (Fig 6, Table 3). Five out of seven groups exhibited a negative difference between Ti50 in TC and CC, indicating a generally faster diffusion in TC compared to CC. On average, the difference between Ti50 in TC and CC was -0.62 days. This value was significantly correlated with male percentage (ρ = -0.78, p-value < 0.05).

**Table 3:** Initial compartment 1 half-emptying factor for all progenies.

Progeny	Ti50 CC (dpf)	Ti50 TC (dpf)	Ti50 TC–Ti50 CC (dpf)	Males (%)
P1S1R1	11.77	10.91	-0.86	12.82
P1S1R2	11.45	11.29	-0.16	12.33
P1S2	12.81	11.56	-1.25	8.51
P2S3	12.53	13.39	0.86	0.69
P3S4	11.93	12.16	0.23	2.33
P4S5R1	13.85	11.69	-2.16	46.39
P4S5R2	13.10	12.08	-1.01	40.4

Diffusion step in terms of dpf at which 50% of the fish left the initial compartment 1 (Ti50: initial compartment 1 half-emptying factor) for each progeny in thermal and control continuums.

## Discussion

*Oreochromis niloticus* has a complex sex determination mechanism with major and minor genetic factors (XX|XY system and polygenic factors) and a temperature effect (GSD+TE) [10,11]. Our experiment was designed to provide some insight into the relevance of temperature effects on Nile tilapia sex determination when fish have the opportunity to choose their thermal preference in a horizontal thermal step gradient, which represents some temperature choices that fish could make at a specific period of their life in the wild.

### Juvenile occupation in the continuum

Our study shows that sexually undifferentiated juvenile tilapia moved across a three-connected-compartment device (continuum). In a control continuum (CC) where temperatures in the three compartments were identically set to 28°C at all times, the 10 dpf juveniles evenly occupied the three compartments after the first 5 days and that distribution stayed essentially even until the end of the experiment (40 dpf). Cerqueira *et al*. (2016) found similar results on 19.2 ±3.2 g tilapia (*O*. *niloticus*) 40 hours after transfer into a multichamber device (control at 26.50 ±0.33°C) with a homogeneous distribution occurring throughout the chambers. A previous study using African catfish (*Clarias gariepinus*) in the same device (step temperature gradient continuum with 3 compartments) similarly showed that undifferentiated juveniles spread nearly evenly across the control continuum during the 12 days of the experiment [29]. Other studies conducted on adult zebrafish (*Danio rerio*) also reported an almost random distribution among chambers [30,31]. These observations are indicative of an explorative behavior by which fish diffuse from the initial compartment and tend to occupy all the available space without any positional preference. No significant difference in the relative occupation pattern in CC was observed when the different periods of the day (morning, midday, evening) were considered separately in the present study.

In the thermal continuum (TC), two occupation patterns were observed during and after the increase in temperature in compartments 2 (32°C) and 3 (36.5°C). During the high temperature treatment period (10 to 29 dpf), fish distribution across compartments was significantly different compared to that in CC. In contrast, during the period from 30 to 40 dpf, where compartment temperatures were equally set to 28°C as in CC, the distribution across compartments was again even and indistinguishable from CC. Taken together, these observations indicate that the occupation pattern observed in TC during the treatment period was due to the higher temperatures in compartments 2 (32°C) and 3 (36.5°C).

The most dramatic illustration of behavioral thermal homeostasis occurs due to viral or bacterial infections, which were shown to modify thermal preferendum in several species. When challenged by *Streptococcus iniae*, Nile tilapia exhibited behavioral fever 24 h postinfection with a shift in its thermal preferendum from 30 to 32°C. A return to normal thermopreference occurred after 4–5 days [32]. In *Cyprinus carpio carpio*, infection by CyHV-3 virus induces a shift to a higher thermal preference (24°C to 32°C) from 4–6 days post infection (dpi) to 13 dpi. This phenomenon called ‘behavioral fever’ was also observed in zebrafish larvae [33]; it helps ectothermic organisms fight infection and reduce mortality [24]. We did not find any negative correlation between compartment 3 (36.5°C) occupation during the high temperature treatment period and survival in TC. A negative and significant correlation was found between occupation in compartment 2 (32°C) and survival rate in the entire TC. However, the complete absence of clinical signs of pathologies during our experiment allowed us to reject the involvement of behavioral fever in the observed thermal preferences.

Another type of stress was reported to induce modification in the thermal preference of fish. Confinement induces hyperthermia in zebrafish expressed as ‘emotional fever’ [34,35]. Netting and restraining fish into a small fishing net for 15 minutes was sufficient to observe a shift to higher temperature preference before returning to the initial, previous preference after 8 hours. To avoid this effect in our experiments, fish were counted, split into different batches, and introduced in compartment 1 one day before starting the experiment at 9 dpf.

Animal personality traits also influence behavior in a context-dependent manner [31,32]. In fish, three personality types have been described: 1) proactive: active coping, risk takers suggesting a higher energy requirement, 2) reactive: passive coping, shy with a low level of aggression and immobility suggesting lower metabolic costs, and 3) intermediate individuals between these two extremes. In Nile tilapia and zebrafish, proactive individuals exhibit a higher thermal preference compared to reactive individuals [31,36]. In our experiments, we used unsorted batches; thus, the distribution of thermal preferendum observed should reflect a mixed personality population. Interestingly, such an interpretation would lead us to conclude that the individuals preferring the higher temperatures, in particular those that prefer 36.5°C during the initial period and later on, would be the most proactive, exploratory individuals.

The diffusion kinetics across the continuums during the first days showed a difference between CC and TC. Five out of seven progenies migrated faster into adjacent compartments with higher temperatures, suggesting that the temperature gradient accelerated the exploratory behavior. A similar observation was made by Cerqueira *et al*. (2016) in Nile tilapia, where latency to exit the initial chamber into a thermal gradient was lower compared to the control situation. Conversely, adult zebrafish revealed no difference in exit latency between control and thermal gradient conditions [31].

Further support for enhanced exploratory behavior comes from our observation that, for a short period during the first five days, the higher temperature compartment (36.5°C) was preferentially occupied at the expense of compartment 1 compared to CC. This preference was consistently observed at all three time points each day during this period (Fig 3B). After that initial period, a second thermal preference at 32°C appeared at 19 dpf that remained until the end of the high temperature treatment and corresponded to the optimal temperature for growth of juvenile Nile tilapia [37]. We consider this the final temperature preferendum (FTP) of Nile tilapia according to the definition given by Fry (1971) of “a temperature around which all individuals will ultimately congregate regardless of their thermal experience before being placed in the thermal gradient”. It is generally assumed that the FTP of fish is the optimal temperature for growth and other physiological functions [19,38,39].

Existence of two successive thermal preferenda in our experiment could be related to the age and size of the fish. The optimal temperature for growth evolves depending on body mass in *Oreochromis aureus*, a species closely related to *O*. *niloticus* [40]. Rey *et al*. (2017) also showed that the thermal preferendum changed according to developmental stages in zebrafish larvae. Another explanation may result from the specific setup of our experiment, with the first shorter period being required for complete exploration of the available options, before settling on the FTP of 32°C. A similar experiment conducted with *Clarias gariepinus* revealed a preference for 32°C during the first 24 h after introduction of the fish into the thermal gradient, compared to a 30°C FTP that was observed after 6 days [38]. Further experiments will be required to clearly address this issue.

Interestingly, we also observed that the FTP was not homogenous during the day. In the morning, fish exhibited a significantly lower thermal preference for 32°C compared with midday and afternoon preferences. Such circadian rhythms of preferred temperatures inside a thermal gradient were previously described in several species [41–43]. In Brown trout (*Salmo trutta*) and goldfish (*Carassius auratus*), a higher thermal preference was found at dusk. The authors suggested that the circadian rhythm of preferred temperature may have physiological and ecological value. Events in nature such as feeding opportunities may differ at different periods of the day and night. Additionally, fish are poikilothermic animals expressing behavioral homeostasis through modification of their temperature preference to adapt their metabolism as needed [19,42].

### Impact of thermal preferendum on sex ratio

Progenies in CC never encountered masculinizing temperatures; thus, the expected sex ratio was 100% females, as observed in 2 out of 5 progenies. The other 3 progenies exhibited a skewed sex ratio toward males (from 2.0 to 16.0%). This unexpected occurrence of males in presumptive purely genetic XX populations indicates that a simple, classical sex determination mechanism model (XX|XY combined with TE) is not sufficient to completely explain sexual determinism in Nile tilapia. Minor genetic factors may play an additional role in sexual determinism [10,44–46]. At this point, we also cannot rule out the possibility that the previous selection of hormonally sex-reversed males might somehow have led to offspring that are more susceptible to masculinization. The involvement of epigenetic mechanisms, such as DNA methylation on cytosines, in sex reversal induced by high temperature was proven in several species, including Nile tilapia [47,48]. In half-smooth tongue sole (*Cynoglossus semilaevis*), pseudomales resulting from thermal sex reversal transmitted epigenetic marks to their offspring, leading to an increased neomale population through spontaneous sex reversal without temperature induction [47].

In the thermal control tank (TCT), all juveniles were reared at high temperature (36.5°C) during the gonadal differentiation period (10 to 30 dpf) to evaluate the sensitivity to thermal sex reversal of the progenies. All progenies used in this study were sensitive to thermal sex reversal, albeit to various degrees (from 25.6 to 96.7%), confirming the previously observed parental effect on intrinsic thermosensitivity in Nile tilapia [10,46].

The thermal choice proposed in the continuum led to a statistically significant increase in the proportion of males, except in the least thermosensitive progeny. We propose calling this phenomenon "behavioral sex reversal". In all TC batches, the male proportion was smaller compared to batches undergoing continuous exposure to 36.5°C (TCT). The duplicated progenies P1S1R1/R2 and P4S5R1/R2, as well as the two spawns from the same couple (P1S1 and P1S2), presented very similar responses in TC and TCT. We found strong positive correlations between male percentages in the 3 environments of CC, TC and TCT. These results indicate that the extent of sex reversal caused by a thermal gradient is linked to the intrinsic thermosensitivity of the progeny. Moreover, the intrinsic thermosensitivity was also linked to the extent of spontaneously appearing neomales without any masculinizing treatment. In conclusion, the amplitude of sex reversal, spontaneously or under high temperature exposure, could be an intrinsic characteristic of a specific couple.

It was previously stated that temperatures above 32–35°C can induce masculinization in Nile tilapia [20,21]. We did not find a correlation between the mean occupation rate of compartment 3 (36.5°C) during the entire treatment period and the percentage of males in the TC batch. Santi *et al*. *(*2017) reported similar results in *Clarias gariepinus*. However, as discussed above, we observed a significant preference for 36.5°C during the first 5-day period at the beginning of thermal treatment in TC, after which the thermal preferendum switched to 32°C until the end of the high temperature period. Baroiller *et al*. (1995) showed that a 10-day treatment period was sufficient to skew the sex ratio toward males, while Rougeot *et al*. (2008) found that a very short high temperature treatment during the embryonic phase (from 12 hours post fertilization pf to hatching) resulted in an increased male proportion. Taken together, these observations suggest that high temperature masculinization could take place inside a thermal gradient during a very short period shortly after 10 dpf. Interestingly, this time point corresponds to the end of the strictly maternal mouth-brooding period, when juveniles are released and can experiment potentially masculinizing temperatures in the shallow margins of water bodies [10,49].

In the present study, we observed that diffusion kinetics in TC varied across progenies. Only two progenies exhibited a slower diffusion in TC compared to CC, corresponding to those presenting no or a small amount of thermal masculinization in TC. In fact, we observed a clear negative correlation between the time needed for 50% of the fish to leave compartment 1 (Ti50) and the masculinization rate in TC. This suggests that the more proactive, exploratory populations may be more prone to exposure to higher temperatures (possibly also exploring compartment 3 more intensely, see above) and would thus experience the highest sex reversal rates.

The overarching question concerning temperature- (or, in general, environmentally) induced sex reversal is whether this phenomenon is actively maintained through evolution in certain species to better cope with potentially dangerous situations or to adapt the sex ratio in changing environments. What could be the selective advantage of sex reversal for the survival of a species? In swordtail (*Xiphophorus helleri*), the appearance in exclusively monosex populations of individuals of the opposite sex [50] clearly will allow this population to procreate. In *Menidia menidia*, a strict ESD species, females are produced at lower temperatures compared to males, earlier in the year, thus giving the females a longer time to grow until reaching the breeding season. This temporally separated generation of the two sexes generates larger females compared to males and largely contributes to the reproductive success of this species [5,51]. For Nile tilapia, unfavorable conditions that may be encountered in their African habitat are high temperatures and drought (more so in the context of global warming). The occurrence of sex-reversed males in natural populations, which never met with any known masculinizing factor, highlights the potential importance of these individuals. Masculinization at high temperature would potentially affect the fecundity of a population. When exposed to high temperatures, partial masculinization of a population would lead to the emergence of XX neomales in the first generation, which also grow faster and may therefore be more fit to survive. Breeding of these XX males with normal females in the following generation would lead to an increase of the female population and thus to an improvement of the reproductive potential of the entire population and a faster recovery of the population [1]. Another advantage in stressful conditions could come from the recombination pattern between chromosomes during meiosis, which is different according to the phenotypic sex. In sex-reversed individuals, unexpected recombinations could occur on sex chromosomes, leading to novel means of genetic mixing [52]. Modification of the temperature environment could also modify growth and survival. It was shown that in blue tilapia (*Oreochromis aureus*), exposure of sexually undifferentiated juveniles to fluctuating temperatures (35°C night and 27°C day and vice versa) skewed sex ratios toward males, induced faster growth, decreased size heterogeneity and allowed for better survival compared to controls reared at a constant 27°C [53].

Our study does not allow confirmation of any of these hypotheses; however, it does show that, when presented with a choice of different thermal conditions during the sex determination period, Nile tilapia juveniles tend to prefer higher temperatures even though they experience masculinization. This indicates that such a behavior is at least not deleterious to the population, otherwise, it would have been avoided. Whether this behavior is actually beneficial for the population remains to be determined. It is interesting to note that our observations show that there is a familial component in the extent of sex reversal observed, as well as a clear link with the exploratory character of a population.

## Conclusions

To conclude, our study shows that fish migration in a horizontal thermal step gradient depends on multiple factors and evolves during the course of the day. In a thermal continuum, undifferentiated juveniles (1) spontaneously migrate to the higher, potentially masculinizing temperatures during the critical period of sex differentiation, and (2) this migration to high temperatures is sufficient to induce variable but significantly skewed sex ratios toward males. The higher the intrinsic thermosensitivity and ability to generate spontaneous sex-reversed males are, the higher the male proportion in the thermal continuum is. Finally, the factors that motivate this preference for high masculinizing temperatures remain to be elucidated.

## Supporting information

S1 Table Male proportion and survival rates. Male proportion (%) and post-treatment survival rates (%) at 40 dpf for all progenies and treatments (control and thermal continuums and thermal control).
(DOCX)
