# Correction: Temperature preference of Nile tilapia (*Oreochromis niloticus*) juveniles induces spontaneous sex reversal

**DOI:** 10.1371/journal.pone.0214689

**Published:** 2019-03-26

**Authors:** Renaud Nivelle, Vincent Gennotte, Emery Jules Kembolo Kalala, Nguyen Bich Ngoc, Marc Muller, Charles Mélard, Carole Rougeot

There are errors the Funding statement. The correct Funding statement is as follows:

RN is a Ph.D. grant holder from FRIA-FNRS (Fond pour la Formation et la Recherche dans l’Industrie et dans l’Agriculture). MM is a "Maître de Recherche" at the "Fonds National de Recherche Scientifique". This study was supported by ULiège-ARC program (Actions de Recherches Concertées, ARC 15/19-7) as well as the University Foundation of Belgium (AS-0265). The funders had no role in study design, data collection and analysis, decision to publish, or preparation of the manuscript.
