# Supplementary material for: Temperature preference of Nile tilapia (Oreochromis niloticus) juveniles induces spontaneous sex reversal
Source: PLoS One. 2019 Feb 14;14(2):e0212504. doi: 10.1371/journal.pone.0212504 (PMC6375642; doi:10.1371/journal.pone.0212504)
Supplement: S1 Table — Male proportion (%) and post-treatment survival rates (%) at 40 dpf for all progenies and treatments (control and thermal continuums and thermal control). (DOCX) [file pone.0212504.s001.docx]

**S1 Table. Male proportion and survival rates.**

| **Progeny** | **Treatment** | **N Sexing** | **N Males** | **N Females** | **% Males** | **N initial** | **N Alive** | **N Death** | **Survival (%)** |
| --- | --- | --- | --- | --- | --- | --- | --- | --- | --- |
| **P1S1R1** | Control Continuum | 100 | 3 | 97 | **3.0** | 150 | 145 | 5 | **96.7** |
| **P1S1R2** | Control Continuum | 102 | 1 | 99 | **1.0** | 150 | 142 | 8 | **94.7** |
| **P1S2** | Control Continuum | 149 | 2 | 143 | **1.3** | 150 | 150 | 0 | **100** |
| **P2S3** | Control Continuum | 71 | 0 | 71 | **0.0** | 150 | 120 | 30 | **80.0** |
| **P3S4** | Control Continuum | 129 | 0 | 127 | **0.0** | 150 | 128 | 22 | **85.3** |
| **P4S5R1** | Control Continuum | 100 | 16 | 84 | **16.0** | 150 | 142 | 8 | **94.7** |
| **P4S5R2** | Control Continuum | 100 | 16 | 81 | **16.0** | 150 | 145 | 5 | **96.7** |
| **P1S1R1** | Thermal Continuum | 117 | 15 | 102 | **12.8** | 150 | 130 | 20 | **86.7** |
| **P1S1R2** | Thermal Continuum | 73 | 9 | 64 | **12.3** | 150 | 103 | 47 | **68.7** |
| **P1S2** | Thermal Continuum | 141 | 12 | 127 | **8.5** | 150 | 143 | 7 | **95.3** |
| **P2S3** | Thermal Continuum | 145 | 1 | 143 | **0.7** | 150 | 145 | 5 | **96.7** |
| **P3S4** | Thermal Continuum | 129 | 3 | 126 | **2.3** | 150 | 129 | 21 | **86.0** |
| **P4S5R1** | Thermal Continuum | 97 | 45 | 52 | **46.4** | 150 | 114 | 36 | **76.0** |
| **P4S5R2** | Thermal Continuum | 99 | 40 | 59 | **40.4** | 150 | 134 | 16 | **89.3** |
| **P1S1R1/R2** | Thermal Control | 100 | 50 | 50 | **50.0** | 294 | 278 | 16 | **94.5** |
| **P1S2** | Thermal Control | 54 | 38 | 16 | **70.4** | 314 | 296 | 18 | **94.2** |
| **P2S3** | Thermal Control | 121 | 31 | 84 | **25.6** | 695 | 612 | 83 | **88.0** |
| **P3S4** | Thermal Control | 120 | 57 | 55 | **47.5** | 513 | 473 | 40 | **92.2** |
| **P4S5R1/R2** | Thermal Control | 61 | 59 | 2 | **96.7** | 157 | 120 | 37 | **76.4** |

Male proportion (%) and post treatment survival rates (%) at 40 dpf for all progenies and treatments (control, thermal continuums, and thermal control).
